# Ibuprofen-Loaded CTS/nHA/nBG Scaffolds for the Applications of Hard Tissue Engineering

**DOI:** 10.29252/.23.3.190

**Published:** 2019-05

**Authors:** Pawan Kumar, Brijnandan S. Dehiya, Anil Sindhu

**Affiliations:** 1 Department of Materials Science and Nanotechnology, Deenbandhu Chhotu Ram University of Science and Technology, Murthal 131039, India; 2 Department of Biotechnology, Deenbandhu Chhotu Ram University of Science and Technology, Murthal 131039, India

**Keywords:** Chitosan, Fibroblast, Ibuprofen, Nanoparticles

## Abstract

**Background:**

This study addressed the development of biodegradable and biocompatible scaffolds with enhanced biomechanical characteristics. The biocompatibility and the cationic nature of chitosan (CTS) make it more effective as a bone grafting material.

**Methods:**

The hydroxyapatite nanoparticles (nHA) were synthesized by hydrothermal method, and bioglass (nBG) (50% SiO_2_-45% CaO-5% P_2_O_5_) was synthesized using sol-gel method. The ibuprofen-loaded CTS/nHA and CTS/nBG scaffolds were fabricated by using freeze-drying method.

**Results:**

Transmission electron microscopy image of nHA and nBG revealed the particles of less than 200 nm. The scanning electron microscopy (SEM) images of CTS/nHA and CTS/nBG scaffolds showed pore sizes ranging from 84-190 µm. The physiochemical characteristics of synthesized ceramic nanoparticles and scaffolds analyzed by XRD were confirmed by ICDD 9-432. The porosity of scaffolds was measured by using SEM, Brunauer-Emmett-Teller method, and Archimedes’ principle. The open porosities of CTS/nBG and CTS/nHA samples were 29% and 31%, respectively. The compressive strength of scaffolds was evaluated by stress vs. strain curve. The CTS/nHA scaffold revealed 4% more water retention capacity than CTS/nBG scaffold. In the presence of lysozyme, CTS/nBG scaffold degraded 32.8%, while CTS/nHA degraded 26.1% in PBS solution at pH 7.4. The density of all scaffolds was found (1.9824 g/cm^-3^ and 1.9338 g/cm^-3^) to be nearly similar to that of the dry bone (0.8-1.2 g/cm^-3^). Fibroblast cells multiplied two times in the sample medium of CTS/nBG after 14 days. After 72 h, CTS/nBG and CTS/nHA scaffolds demonstrated 52% and 46% drug release, respectively.

**Conclusion:**

Based on our findings, ibuprofen-loaded scaffolds could be an effective drug delivery system for tissue engineering applications.

## INTRODUCTION

Trauma, injury, infections, and degenerative diseases can lead to bone loss as a health threatening problem. Tissue Engineering, as a promising technology, can provide new treatment opportunities with eliminating issues like donor site morbidity, less availability of tissue donor, immunogenicity, and infection-causing microbes transfer. The pore sizes of human bones, including long, short, flat, and irregular are 100-300Z μm[1]. Nutrients can actively diffuse from bone-blood supply to the bone tissue via 150-200 μm pores. Bone tissue also contains mineralized (type-1 collagen) and non-mineralized organic components (4 nm thick carbonated apatite) as a part of the bone extracellular matrix (ECM)[2,3]. In bone tissue engineering, artificial bone graft materials such as inorganic materials (hydroxyapatite nanoparticles [nHA], bioactive glass, etc.) and biodegradable polymers [poly(lactic acid), poly(lactic-co-glycolic) acid, gelatin, and soon] have been used as substitutes. However, none of the above materials can meet all the demands because of low mechanical properties[4,5]. Engineered biomaterials might cause chronic post-surgical paint that is attributed due to changes in the central nervous system arising because of nerve injury/inflammation. To reduce pain and excessive inflammation, oral administration of nonsteroidal anti-inflammatory drugs is very common due to their lesser side effects compared to opioids[6]. However, the large dose of ibuprofen may produce adverse effects such as gastric ulcer and renal failure. This situation justifies the significance of ideal drug delivery in tissue engineering. Anti-inflammatory drug delivery supports new tissue generation without pain and inflammatory reactions. Tissue engineering also includes the injection of specific cells in an injured tissue, delivery of different biomolecules such as polysaccharides, growth factors, and peptides to a selected tissue, which support growth and regeneration of injured tissues[7]. Chitosan (CTS) is a derivative of naturally occuring polymer chitin, used in tissue restoration[8]. Positive charged CTS can bind to negatively charged molecules (glycosaminoglycan [GAG]) and proteoglycans[9]. GAG is the major component of the ECM of bone and cartilage, and polysaccharide backbone of CTS is structurally similar to GAG[10]. GAG is also the main component of ECMs in tissue and causes cell adhesion and proliferation due to its high biocompatibility and non-immunogenic properties. The CTS can be used to form different porous three dimensional structures[11], gels[12], thin films[13], membranes[14], and fibers[15], which all are very promising for bone tissue regrowth. However, CTS has some downsides, such as lack of sufficient mechanical strength, rapid degradation, and lacking of bioactive cell signaling molecules, which are vital for the regeneration of bone tissue[16].

The nHA showing good phase and dimensional stability. It will significantly improve composite density, smoothness, and fracture toughness. Due to the inert property, nHA have medical and health applications; they are present in the inorganic part of bones and human teeth as well as in cuttlefish shells and corals. The equivalent crystal size identified in chemically synthesized HA and natural HA can be seen in hard tissues. In our previous investigation on HA, we have observed the standard stoichiometric ratio of 1.667 (Ca/P)[17]. HA boosts up the formation of apatite-like structure between HA and tissues by supporting chemical interactions[18,19].

Bioglass is a bioactive material that induces biological activity by promoting some surface reactions. It supports the formation of apatite-like structure between bioglass and hard tissues when implanting in the human body. The formation of apatite layer confirmed the effective biological interaction and fixation of hard tissue with the material surface[20]. The apatite layer can be generated artificially on the surface of bioglass when the material is placed in the simulated body fluid (SBF) for some days. The main goal of this study was to fabricate ibuprofen-loaded CTS/nHA and CTS/nBG (bioglass nanoparticles) scaffolds with improved mechanical and biological properties. Loading of these two new compositions (CTS/nHA and CTS/nBG) by ibuprofen may help in the restoration of tissue defects.

## MATERIALS AND METHODS

Acetic acid (SUK-320099), sodium hydroxide pellets (SUK-567530), low molecular weight CTS (75-85% deacetylated, SUK-448869), glutaraldehyde (SUK-G5882), calcium nitrate tetrahydrate (SUK-C1396), phosphorus pentoxide (SUK-431419), diammonium hydrogen phosphate (SUK-1012070500), tetraethyl orthosilicate (SUK-131903) and ibuprofen salt (SUK- I1892) were procured from Sigma Aldrich (USA). Lysozyme (code 45822) was purchased from Sisco Research Laboratories Pvt. Ltd (India).

### Synthesis of hydroxyapatite and bioglass nanoparticles

**Fig. 1:**
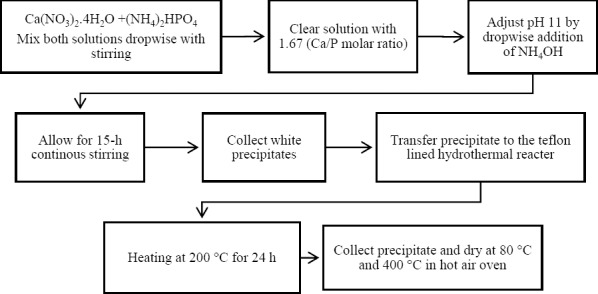
Hydroxyapatite nanoparticles (nHA) synthesis using hydrothermal method.

The stepwise hydrothermal synthesis of nHA is shown in Figure 1. At first, 1 M solution of both calcium nitrate tetrahydrate (11.807 g) and diammonium hydrogen phosphate (6.603 g) was prepared using 50 mL distilled water and subsequently diluted further to create 0.10 M solutions. By the drop-wise addition of the calcium nitrate tetra hydrate solution (50 mL) to the di-ammonium hydrogen phosphate solution, precipitate was formed with continuous stirring to get a 1.67 (Ca/P ratio) in the miscellaneous solution[21]. The white colored crystalline powder was collected after drying at 80 °C and 400 °C.

The sol-gel method was selected for the synthesis of nBG. For the synthesis of the novel composition of bioglass (50% SiO_2_-45% CaO-5% P_2_O_5_), 8.108 g of tetraethyl orthosilicate was mixed with 100 ml of ethanol and allowed continuous stirring for one hour. After that, 4.454 g of calcium nitrate tetrahydrate and 0.534 g of phosphorus pentoxide were dissolved in distilled water with 30 minutes stirring to get 100 ml of mixture, which was added dropwise to tetraethyl orthosilicate solution until a homogenous mixture with pH 5 was obtained. Ammonia solution was used to maintain pH at 11, and the solution was transferred to an incubator and kept for 72 h for aging in order to obtain the gel. The aged gel was placed in an oven at 100 °C for drying to eliminate ethanol[22,23]. The dried gel was then heated at 700 °C for 24 h to stabilize the glass and also to eliminate residual nitrate[24].

### Scaffolds synthesis

**Fig. 2:**
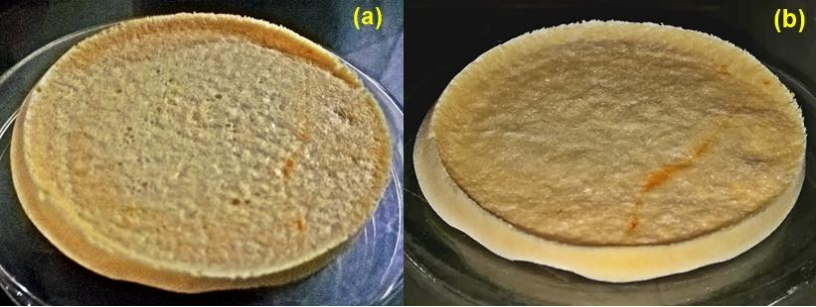
Images of ibuprofen-loaded CTS/nBG (a) and CTS/nHA (b) scaffold.

Ibuprofen loaded- and unloaded-CTS/nHA and -CTS/nBG scaffolds were synthesized using freeze-drying method. Acetic acid (1% w/v) was mixed with 2% (w/v) CTS solution (75-85% deacetylated). Glutaraldehyde (0.25%) was incorporated in the mixture solution that helps to make cross-linking under agitation at room temperature for 15 h. An appropriate amount of nHA and nBG was added to the mixture solution separately with continuous 15 h stirring for proper mixing. Then ibuprofen (10% w/w of polymer) solution was added to the mixture solution separately[25,26], and the homogeneous mixture of ibuprofen-loaded and -unloaded samples were distributed into Petri dishes. The dishes were placed within a deep freezer at -80 °C. The frozen sample was kept at lower temperature in a chamber where the ice was removed by sublimation and the unfrozen water by desorption in a secondary drying process[27]. The unwanted acetate was removed by using 10% NaOH solution, followed by three times washing with distilled water. The lyophilizer was used for 48 h to lyophilize the frozen hydrogels. Sodium borohydride solution (5%) and deionized water were used to block non-responded aldehyde group in the samples[8,28,29]. The scaffolds of ibuprofen-loaded-CTS/nBG and -CTS/nHA are shown in Figure 2.

### Standard calibration curve of ibuprofen

**Fig. 3:**
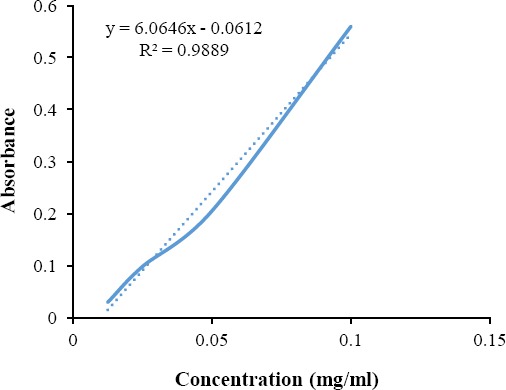
Standard calibration curve of ibuprofen.

The stock solution of ibuprofen was prepared by dissolving 20 mg drug into 100 ml phosphate buffer (pH 6.8), which was used to plot a standard curve of ibuprofen. The four different concentrations (0.1, 0.05, 0.025, and 0.0125 mg/ml) were prepared from the stock solution. UV-visible spectrophotometer (Shimadzu, Japan) was used to analyze the absorbance of these dilutions. The standard curve was prepared by plotting different concentrations of ibuprofen against absorption as shown in Figure 3.

### Measurement of ibuprofen release

**Fig. 4:**
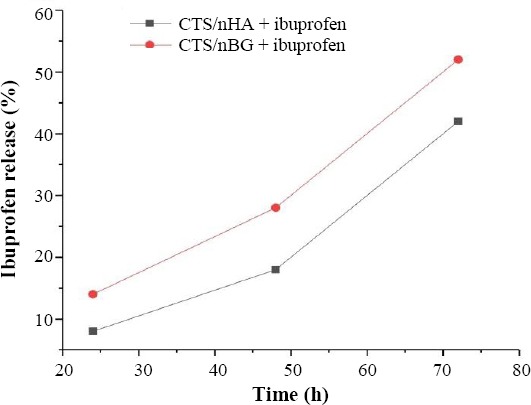
Ibuprofen release with respect to time changes.


The drug-loaded scaffolds were cut into small pieces of 25 mg and placed into the PBS at 37 °C. The drug release from scaffolds was monitored after 24 h, 48 h, and 72 h using UV/Vis spectroscopy at 220 and 218 nm. The results of UV/Vis spectroscopy were calibrated with the calculated standard curve of ibuprofen[25] and are shown in Figure 4. The amount of ibuprofen release was estimated by equation 1. Experiments were run in triplicates.

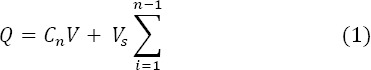


Where Q is the amount of ibuprofen released, C_n_ is the concentration of samples, V is the volume of *in vitro* released solution, and V_s_ is the sampling volume.

### Characterization

#### XRD analysis

The samples of nanoparticles (nHA and nBG) as well as ibuprofen-loaded and -unloaded scaffolds (CTS/nBG and CTS/nHA) were analyzed with XRD (Rigaku Ultima IV) with the scanning rate of 1° per second over the range of 2θ angles from 15° to 60°.

#### Transmission electron microscopy (TEM)

TEM produces high-resolution images by transmitting high-energy electron through the specimen. Structure, composition, and size of nanoparticles (nHA and nBG) were analyzed by TEM (Hitachi H-7500, Japan);

#### Scanning electron microcopy (SEM)

The detailed surface information such as composition, topography, and porosity of samples were observed by scanning electron microscopy (SEM; JEOL, Japan). The surface and fracture section of the samples were coated with gold.

### Porosity

The pore volume, micro-pore radius, and pore specific surface area of the sample were examined by BET (Quantachrome® Nova Station, USA). The small sized pieces of the scaffold were located in the sample tube, and the measurement conditions were set. Most of the results were concentration and viscosity dependent.

### Mechanical properties

**Fig. 5:**
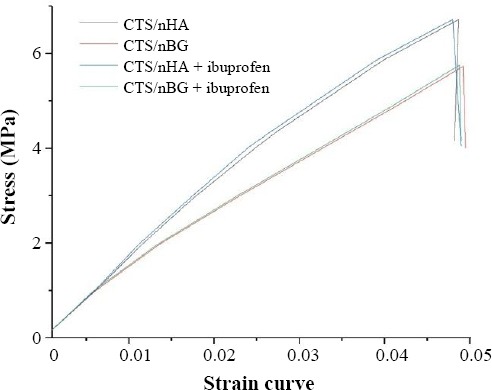
Stress vs. strain curve of ibuprofen-loaded and -unloaded scaffolds.

The sample size of 4 × 4 × 4 mm^3^ was cut from the scaffolds for the compressive strength measurement. The test piece (scaffold) is compressed between the platens of a machine by a gradually applied load. The quantitative results are presented as the mean ± standard error. The stress vs. strain graph was used to explain the compressive strength of the scaffolds (Fig. 5 and Table 1).

**Table 1:** Elastic modulus and compressive strength of scaffolds

Sample	CTS/nHA (MPa)	CTS/nHA + ibuprofen (MPa)	CTS/nBG (MPa)	CTS/nBG + ibuprofen (MPa)
Elastic modulus	±136.5	±138.52	±117.55	±118.58
Compressive strength	±6.69	±6.67	±5.76	±5.74

### Density and open porosity

The open porosity of sample scaffold was calculated by Archimedes’ principle. The small pieces of scaffold were employed for the measurement. The sample was dipped into a water-containing beaker and kept inside the desiccator for 30 minutes. The suspended weight and soaked weight of sample scaffold were taken for further calculations. The equations 2 and 3 used to measure the bulk density and porosity are given below[30]:


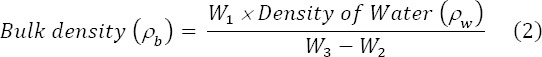



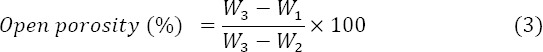


Where W_1_ is the weight of sample in air, W_2_ is weight of sample in distilled water, and W_3_ is wet mass of sample after removal from distilled water.

### Swelling behavior and degradation

**Fig. 6:**
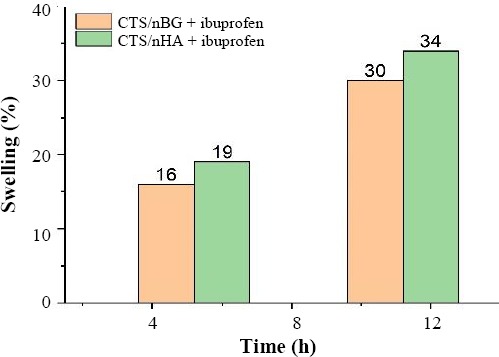
Swelling behavior of scaffolds in PBS.


PBS solution was used to check the swelling or water retention capability of the sample scaffold. The swelling capacity depends on the porosity of the sample and nature of materials. The swelling capacity of the sample was calculated by the equation 4[28,31]. The comparative analysis of swelling behavior of scaffolds is shown in Figure 6.

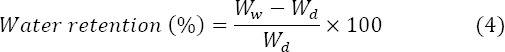


Where W_d_ is initial weight, and W_w_ is the weight of the sample after swelling.

**Fig. 7:**
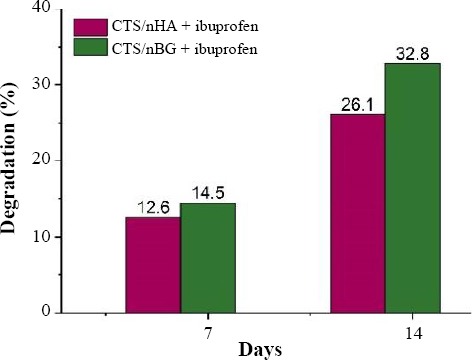
*In vitro* degradation of scaffolds biodegradation.


The measurement of *in vitro* weight degradation of the scaffolds was required to estimate the bioavailability of materials. The pieces of the sample were incubated in the PBS solution (pH 7.4) containing 1 × 10^4^ U/ml of lysozyme at room temperature for 14 days. After the interval of 7 and 14 days, degradation of the sample was recorded by using equation 5[28]. The *in vitro* weight loss comparison between scaffolds is shown in Figure 7.

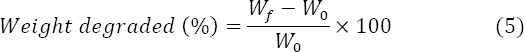


Where *W_0_* is initial weight and *W_f_* is the final weight after degradation sample.

### Mineralization behavior

Every material has distinct bioactivity or the ability to make bonding with body tissues. The bioactivity of any material depends upon various factors, including physiochemical behavior and morphological characteristics of materials. The SBF was used to measure the mineralization behavior of ibuprofen- loaded CTS/nHA and CTS/nBG scaffolds because SBF consists of nearly the same concentration of inorganic constituents in blood plasma.

### Cell studies

To check the cytotoxicity of scaffolds, fibroblast cell lines were maintained in the cell culture facility in minimum essential medium (MEM) with 10% FBS and 100 U/ml penicillin-streptomycin. Before cell seeding, all the sample scaffolds were sterilized and placed in an incubator with cell culture for two hours with 5% CO_2_ and 85% humidity.

The detached cells (1 × 10^5^ cells/100 µl) were seeded dropwise on the surface of scaffolds for the investigation of cytocompatibility. The cell-seeded scaffolds were placed in a humidified incubator at 37 °C for 4 h for the cell attachment.

## RESULTS AND DISCUSSION

**Fig. 8:**
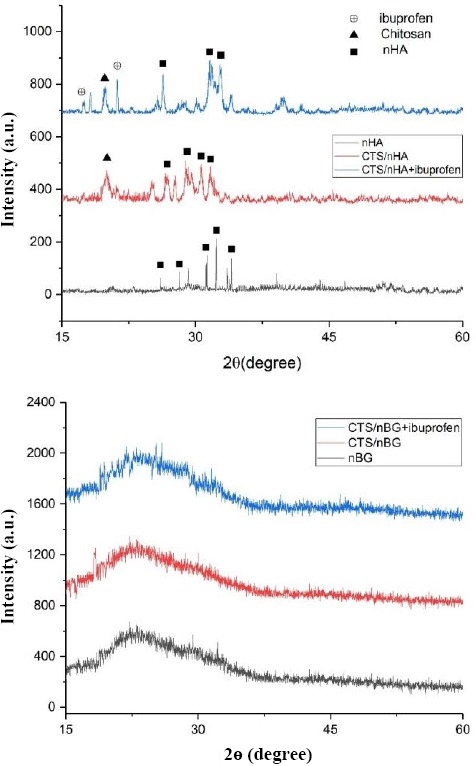
XRD analysis of (A) nHA, CTS/nHA, and CTS/nHA + ibuprofen samples and (B) XRD analysis of nBG, CTS/nBG, and CTS/nBG + ibuprofen samples.

**Fig. 9:**
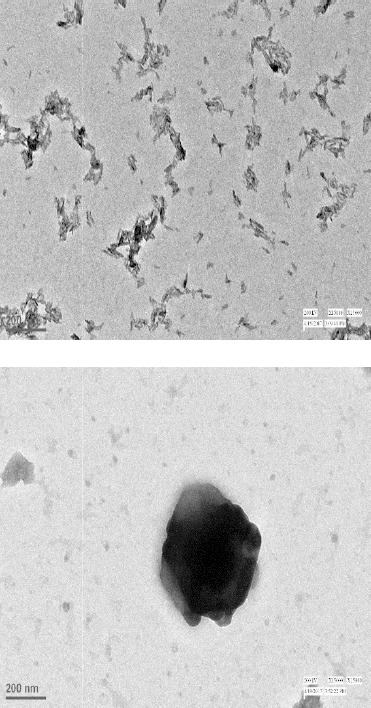
TEM images of (A) hydroxyapatite and (B) bioglass nanoparticles.

**Fig. 10:**
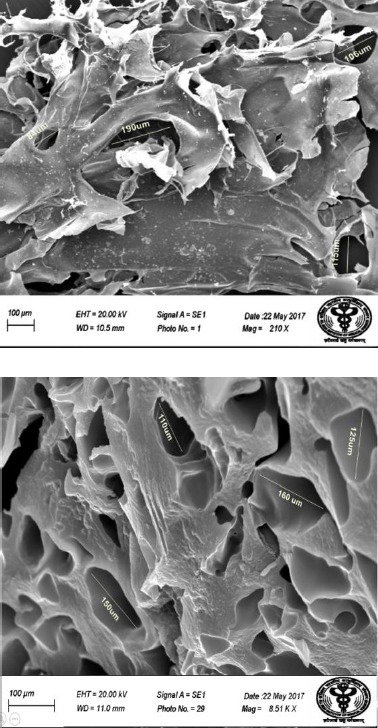
SEM images of (A) CTS/nHA + ibuprofen scaffold and CTS/nBG + ibuprofen scaffold

In this study, white colored hydroxyapatite and nBG were synthesized by hydrothermal and sol-gel method, respectively. Ibuprofen-loaded and -unloaded CTS/nHA and CTS/nBG scaffolds were successfully fabricated by freeze-drying method. The XRD pattern confirmed the fabrication of nanoparticles and scaffolds calibrated with ICDD 9-432. The sharp peaks in spectra verified the crystalline nature of HA, and broad peaks approved the amorphous nature of bioglass. The phase changes in XRD spectrum were observed due to the addition of CTS and ibuprofen as shown in Figure 8. Ibuprofen binds CTS through electrostatic and via hydrophobic interactions, as well as hydrogenbonding[31]. The ammonium groups of CTS (low molecular weight) were involved in ionic interaction with the carboxylate anion of ibuprofen[32]. Bone is composed of 43% minerals (mainly HA, 69-80% of mineral content of bone), 28-30% collagen, 5-7% bone cells, and 10-20% water[27]. The complex structural composition of bone needs a composite-based substitute/implant for bone tissue engineering. The CTS/nHA scaffold consists of nHA (Ca/P) as well as the polymeric phase that can easily mimic the ECM of bone. In CTS/nBG scaffold, nano-silica supports *in vitro* and *in vivo* cell proliferation[10] without toxicity and inflammation[33]. The *in vitro* and *in vivo* tissue ingrowth has been shown in numerous studies[34-37]. CTS can be easily degraded by lysozyme[38] and possesses immunological activity, by activating macrophages, which prevent infections[39,40]. The TEM images revealed irregular shaped nHA and nBG that are illustrated in Figure 9. The SEM image (Fig. 10) of CTS/nBG and CTS/nHA scaffolds showed well-interconnected, heterogeneous pore microstructures. Stretched pores were generated in the scaffold during lyophilization whose formation might be due to hydrogen-bonding formation between polymer and nanoparticles and parallel ice crystal growth. SEM images of scaffolds revealed interconnected mixed size of pores, mainly ranging from 84-190 μm (CTS/nBG) and 110-160 μm (CTS/nHA), which were more relevant for tissue engineering because the pore sizes of bone, muscle, and skin vary from 20-300 μm[17]. The pore size lower than 300 µm contribute to proliferation of osteoblast cell easily through the scaffold[41,42]. Larger pores allow direct osteogenesis and high oxygenation, whereas smaller pores favors osteochondrial ossification[10]. The porous structure of scaffolds provides space for the intracellular matrix formation. Interconnected pores helps to inflow the nutrients and elution of metabolic waste from scaffolds[43].

According to Archimedes’ principle, CTS/nBG scaffold shows 29% porosity, while CTS/nBG scaffold indicates 31% porosity. The surface area and closed porosity of scaffolds were examined by Brunauer-Emmett-Teller (BET), as shown in Table 2. The increase in surface area will allow more contact between body fluid and scaffolds. The higher specific area also affects the release kinetics of drug. Many properties like degradation, swelling behavior, nutrients, or mineral exchange depend on the porosity of scaffolds. The scaffold pore size has an impact on both cell migration and diffusion through the scaffold environment.

**Table 2:** Surface area and porosity measurements of scaffolds

Surface no.	Scaffolds types	Specific surface area (m^2^g^-1^)	Pore specific surface area (m^2^g^-1^)	Pore volume (cm^3^g^-1^)	Micro pore diameter (nm)
1	CTS/nBG + ibuprofen	2.5646	1.9986	0.0030	2.77
2	CTS/nHA + ibuprofen	2.7112	2.1885	0.0052	2.54

**Fig. 11:**
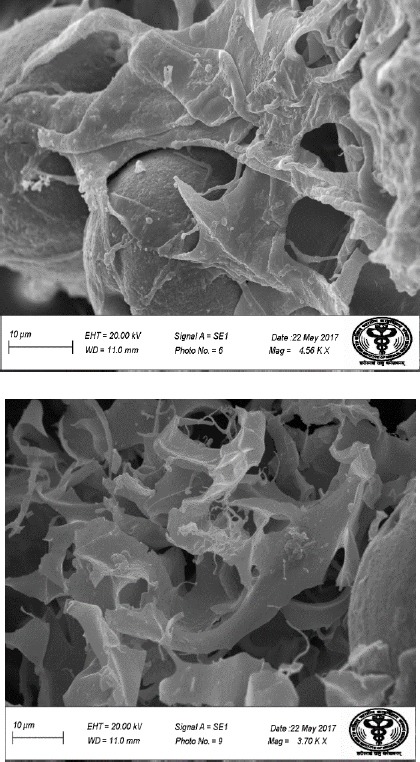
Mineralization of ibuprofen-loaded CTS/nBG (A) and CTS/nHA (B) scaffolds.

From BET results, it was observed that drug-loaded CTS/nBG scaffolds offered comparatively more surface area than CTS/nHA scaffolds. The addition of bioglass to CTS leads to the formation of pores with smaller diameter[40]. The incorporation of ibuprofen in both samples increased the intermolecular networking with CTS[30], which may affect the density and porosity of scaffolds. The density of ibuprofen-loaded CTS/nBG (1.9824 g/cm^3^) and CTS/nHA (1.9338 g/cm^3^) scaffolds is better than CTS and CTS /gelatin scaffolds[44] and comparatively more than the normal dry bone (0.8-1.2 g/cm^3^) and soft tissues (1.01-1.06 g/cm^3^). The density of CTS is very low (0.15-0.30 g/cm^3^) so that bioglass (1-2.6 g/cm^3^) and HA (2-6 g/cm^3^) are selected as a dopant for scaffold fabrication. The addition of nBG and nHA enhanced the density of scaffolds[10,44]. The ibuprofen (10% w/w of CTS) release rate was measured after 24 h, 48 h, and 72h using UV-Vis spectroscopy. All the readings were compared with the calibrated standard curve of ibuprofen. The ibuprofen release profiles for CTS/nBG and CTS/nHA scaffolds showed a slow release of drug after first 24 h, which may be due to the complex structure of scaffolds[43]. The degradation or mineralization behavior of scaffolds may affect the drug release. Drug release approximately 52% corresponds to CTS/nBG and 46% corresponds to CTS/nHA scaffold after 72 h. The CTS/nBG scaffold showed slightly fast drug release as compared to CTS/nHA scaffold. Overall, none of scaffold indicated 100% drug release. The bioglass and HA showed higher absorption, while ibuprofen demonstrated less absorption at 220 nm. Due to high degradation and adsorption capacity of CTS/nBG scaffold, it displayed rapid drug release. The water retention also depends upon porosity or the surface area of scaffolds. The water retention capacity of CTS/nHA scaffold was 34% that is more than CTS/nBG, CTS, and CTS/gelatin[26] scaffolds. The strong interaction between nBG and CTS decreases the swelling rate of scaffolds[10]. The incorporation of ibuprofen in scaffolds may reduce the water retention capability because ibuprofen is non-polar and insoluble in water. Water retention or swelling behavior of scaffolds helps to fill a specific tissue defect and is helpful for the stable implantation[45]. The pre-freezing temperature may affect the physiochemical state of the scaffolds. The *in vitro* weight degradation of CTS/nHA scaffold was found to be 12.6% after seven days and 26.1% after 14 days, while CTS/nBG scaffold was observed to be 14.5% after seven days and 32.8% after 14 days of incubation. The degradation rate of CTS/nBG scaffold was slow due to the neutralization of the acidic degradation products of CTS by the alkali groups leaching from bioglass[10,46]. The degradation rate of CTS/nHA scaffolds decreased when compared to other scaffolds, which may be due to the high degree crystallinity of nHA. The degree of crystallinity and the nature of polymer may also control the hydrolysis and degradation rate[10]. The addition of ibuprofen did not affect the degradation of scaffolds. The regeneration of new tissue needs to replace and degrade the implanted scaffold because the degradation rate plays a crucial role in tissue engineering. The scaffolds were immersed in the beakers containing SBF in an incubator at 37 °C for seven days to check the bioactivity. SBF provides the same environment as blood plasma where scaffolds show slow release of ions in the solvent. The surface dissolution starts mineralization that generates apatite confirmed by SEM (Fig. 11).

**Fig. 12:**
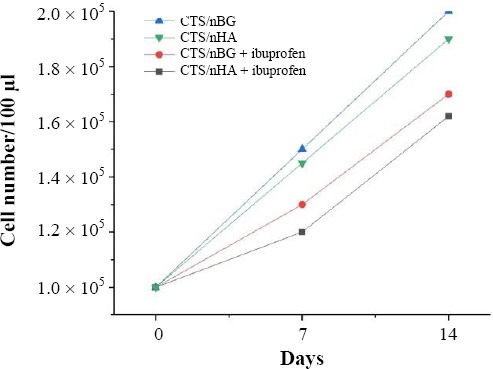
Cell proliferation on CTS/nHA and CTS/nBG scaffolds as a function of time.

The doping of nHA and nBG with CTS made effective improvements in the strength of scaffolds. Ibuprofen loading in the scaffolds made small but significant changes in the compressive strength. CTS/nHA scaffold is more brittle than CTS/nBG scaffold. The incorporation of ibuprofen in scaffolds did not show any influence on fibroblast proliferation[47]. After pre-selected time intervals (7 and 14 days), the number of cells increased (Fig. 12). The CTS/nHA and CTS/nBG scaffolds support that the differentiation of fibroblast cells is faster than ibuprofen-loaded scaffolds, though fibroblast cells were successfully grown on the surface of ibuprofen-loaded scaffolds.

We successfully fabricated ibuprofen-loaded natural polymer based on scaffolds with improved bio-mechanical properties. These scaffolds proved their biocompatibility with fibroblast cells without producing cytotoxicity. Doping of nHA and nBG improved many characteristics of scaffolds such as biocompatibility, biodegradability, swelling, and mechanical behavior etc. The addition of ibuprofen does not affect the cell proliferation activity of scaffolds. The density of both scaffolds meets the parameters of dry bone density. The highly porous structure of scaffolds provides high surface area for cell attachment and nutrient exchange. This structure mimics the functions of the extracellular matrix of bones. The improved mechanical properties of CTS/nHA and CTS/nBG scaffolds will help to create temporary products for tissue engineering. These encouraging results support the potential applications of ibuprofen-loaded CTS/nHA and CTS/nBG scaffolds, as an upgraded substitute, to other natural polymer-based scaffolds for the applications of tissue engineering. The ibuprofen-loaded scaffolds may improve the clinical applications of tissue engineering.
